# Mitochondrial genomes of praying mantises (Dictyoptera, Mantodea): rearrangement, duplication, and reassignment of tRNA genes

**DOI:** 10.1038/srep25634

**Published:** 2016-05-09

**Authors:** Fei Ye, Xu-e Lan, Wen-bo Zhu, Ping You

**Affiliations:** 1 Co-Innovation Center for Qinba Regions’ Sustainable Development, College of Life Science, Shaanxi Normal University, Xi’an, 710062, China

## Abstract

Insect mitochondrial genomes (mitogenomes) contain a conserved set of 37 genes for an extensive diversity of lineages. Previously reported dictyopteran mitogenomes share this conserved mitochondrial gene arrangement, although surprisingly little is known about the mitogenome of Mantodea. We sequenced eight mantodean mitogenomes including the first representatives of two families: Hymenopodidae and Liturgusidae. Only two of these genomes retain the typical insect gene arrangement. In three Liturgusidae species, the *trnM* genes have translocated. Four species of mantis (*Creobroter gemmata, Mantis religiosa, Statilia* sp., and *Theopompa* sp.-HN) have multiple identical tandem duplication of *trnR*, and *Statilia* sp. additionally includes five extra duplicate *trnW*. These extra *trnR* and *trnW* in *Statilia* sp. are erratically arranged and form another novel gene order. Interestingly, the extra *trnW* is converted from *trnR* by the process of point mutation at anticodon, which is the first case of tRNA reassignment for an insect. Furthermore, no significant differences were observed amongst mantodean mitogenomes with variable copies of tRNA according to comparative analysis of codon usage. Combined with phylogenetic analysis, the characteristics of tRNA only possess limited phylogenetic information in this research. Nevertheless, these features of gene rearrangement, duplication, and reassignment provide valuable information toward understanding mitogenome evolution in insects.

The metazoan mitochondrial genome (mitogenome) is an ideal model system for comparative and evolutionary genomic research. The typical mitogenome of metazoans encodes a conserved set of 37 genes for 13 protein-coding genes (PCGs), two ribosomal RNA (rRNA) genes, and 22 transfer RNA (tRNA) genes^1^, with genome-level characters, such as genome size, gene content, and gene order, display high diversity in some lineages^2^,^3^. Gene rearrangements are observed frequently in some groups, while gene duplication and loss are distributed sporadically in limited lineages such as Bivalvia, Cephalopod, and Afrobatrachia^4^,^5^,^6^. These remaining duplicate genes and pseudogenes represent important data for exploring the evolutionary history and mechanisms of gene rearrangement and recruitment. For the arrangement of mitochondrial genes, the variation in relative positions of PCGs and rRNA genes are more limited compared with that of tRNA genes across organisms within a phylum^7^. The tRNA genes with characteristics of diverse changes in relative position, gene content, and secondary structure, are considered as an important tool in studying the evolution of mitogenome, in particular to the rearrangement mechanism^8^,^9^,^10^. Additionally, its variation is usually linked to evolutionary relationships in a wide range of lineages at different taxonomic levels suggesting these features of tRNA could be utilized as useful phylogenetic markers^11^.

The extensive gene rearrangements (including PCGs and RNA) of insect mitogenomes have been detected in several lineages within the Diptera (Trichoceridae, Cecidomyiidae), Hemiptera (Enicocephalidae), Hymenoptera, Thysanoptera, Psocoptera and Phthiraptera^12^,^13^,^14^,^15^,^16^,^17^,^18^, while most of investigated mitogenomes share the same gene order with the hypothesized ancestral pancrustacean mitogenome arrangement^19^ or possess rare tRNA rearrangement. Previously reported dictyopteran mitogenomes consistently display the typical ancestral gene order and content, however only two species are praying mantises and the rest are cockroaches and termites. Members of the Mantodea, a separate lineage within the Dictyoptera, have evolved many unique morphological and behavioural features as the ambush and pursuit predators^20^,^21^,^22^. A better understanding of the diversity of mitogenome evolution in this enigmatic order underlines the need for exploring more taxa with the diverse praying mantis.

Herein, we report eight new mitogenomes from Mantodea and describe their general characteristics. Two new gene rearrangements and reassignment of tRNA genes are described, and evolutionary mechanisms for the gene rearrangements and duplication are discussed. Further, we examine the relationship between tRNA gene duplication and codon usage, and investigate whether these tRNA features vary with phylogeny.

## Results

### General features of Mantodea mitogenomes

**Figure 1:**
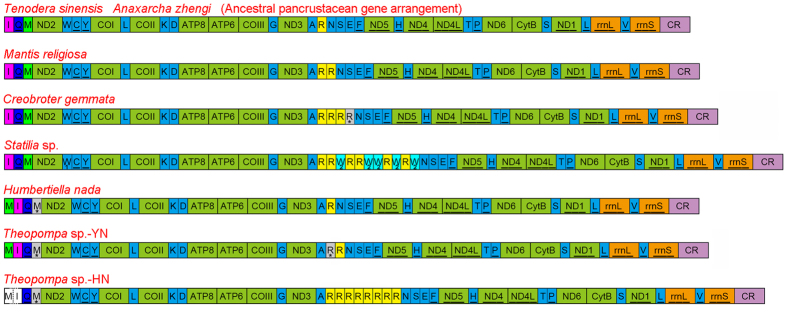
The gene map of the mitochondrial genomes for eight praying mantises. Gene and genome sizes are not to scale. All genes without underline are transcribed in the direction form left to right, and with underline are transcribed in the direction form right to left. Genes labeled an asterisk are pseudogenes.

Seven complete and one nearly complete praying mantis mitogenomes, including the first representatives from Hymenopodidae and Liturgusidae, were generated. The complete mitogenomes are typical circular DNA, and most of the common characteristics of insect mitogenomes are in the Mantodea mitogenomes (Supplementary Table S1–8). Standard collection of 37 mitochondrial genes including 13 protein-coding genes, 22 transfer RNA genes, two ribosomal RNA genes and an A + T-rich region were detected in these mitogenomes, whereas the additional tRNAs were found in four species (*Creobroter gemmata, Mantis religiosa, Statilia* sp., and *Theopompa* sp.-HN). The lengths of these new mitogenomes range from 15,531 bp in *Tenodera sinensis* (Mantidae) to about 17,370 bp in *Theopompa* sp.-HN (Liturgusidae). Although the length variation of A + T-rich region is the main factor for difference in mitogenome size, some extra tRNA gene contents and non-coding regions also contribute to genome length variation. Most of new and published mantodean mitogenomes retain typical insect gene arrangement without considering the multiple gene copies in the original position. However, some gene orders of tRNAs in *Statilia* and Liturgusidae species depart from the common arrangement (Fig. 1).

All analyzed mantodean mitogenomes show base composition biases (Table 1). The range of mantodean A + T content is from 70.1% in *Humbertiella nada* to 77.8% in *Anaxarcha zhengi*, with a mean of 74.2%. Overall, the A + T contents of Liturgusidae species are lower than that of other analyzed species. The distribution of A + T content changed based on various gene or region. For Liturgusidae species, PCGs show the lowest A + T content, while the highest A + T content presents in different region among these three species. The remaining six mitogenomes are under the similar distribution trend in that rRNA and A + T-rich regions possess higher A + T content than PCGs and tRNA, the exception being *Tamolanica tamolana*.

In mantodean mitogenomes, all inferred start codons are canonical start codons ATN except for *ATP6* and *COI. ATP6* of *C. gemmata* uses GTG as the start codon which is accepted conventional start codon for invertebrate mitogenome^23^. *COI* begins with another accepted start condon TTG in *M. religiosa, C. gemmata, Statilia* sp. and *A. zhengi*. For three Liturgusidae species, the *COI* start codon was inferred as non-canonical start codon CTG. Although several candidate common start condons were found around this region, the alignment of amino acid sequences with other mantis species suggests that the triplet CTG could be the start codon for these *COI*. Stop codons are more conserved than start codons in mantodean mitogenomes. Most PCGs invariably end with the typical stop codon TAA or truncated codon TA/T, while limited PCGs (*Theopompa* sp.-YN *COI* and *T. tamolana ND3*) exhibit TAG as stop codon. Comparative analyses of the relative synonymous codon usage of nine mantodean mitogenomes show the similar pattern for codon usage bias (Supplementary Table S9). In particular, the codon usage for Arginine and Tryptophan encoded with multiple tRNA copies is also analogous among their relative species (Table 2).

### Non-coding regions

**Figure 2:**
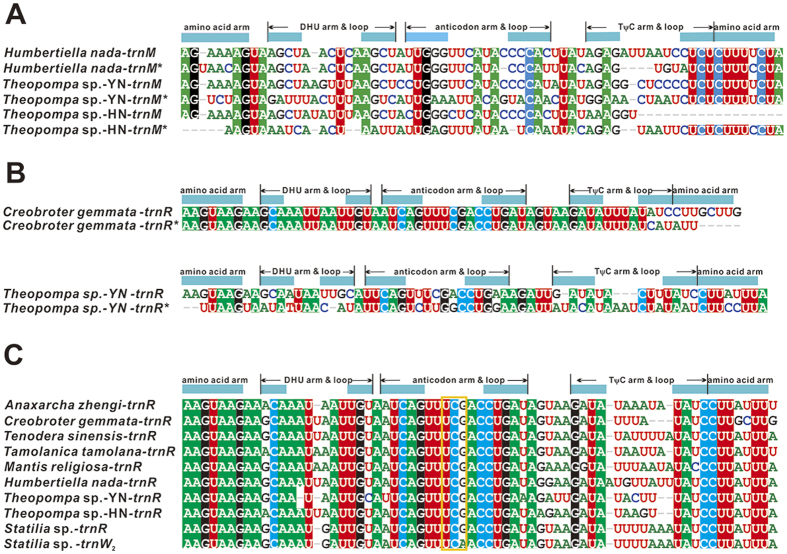
(**A**) Alignment of *trnM* and pseudogene *trnM* sequences in *Humbertiella nada*, and *Theopompa* spp. (**B**) Alignment of *trnR* and pseudogene *trnR* sequences in *Creobroter gemmata* and *Theopompa* sp.-YN. (**C**) Alignment of *trnW*_2_ in *Statilia* sp. and *trnR* in nine praying mantises. Genes with an asterisk are pseudogenes.

In general, insect mitogenomes are highly compact with few short non-coding fragments except for the A + T-rich region. Most of sequenced mantodean mitogenomes share this feature; however, three Liturgusidae species contain 129–138 bp intergenic spacers between *trnM* and *trnI* (this part was not amplified in *Theopompa* sp.-HN) and 59–68 bp intergenic spacers between *trnQ* and *ND2*. Perhaps the origin of these inserts involved gene rearrangement of this tRNA cluster. Indeed, we detected the remnant of *trnM* from the inserts between *trnQ* and *ND2* (Fig. 2A). But in the former inserts, none of homologous fragments of mitochondrial genes or regions were found. In *Theopompa* sp.-YN and *C. gemmata*, 69 bp and 68 bp intergenic spacers were found between *trnA* and *trnR*, and *trnR* and *trnN*, respectively. And these intergenic spacer sequences display high sequence similarity with *trnR* (Fig. 2B). The *T. tamolana* mitogenome also contains a relatively large non-coding sequence (297 bp) between *trnM* and *ND2* including the tandem duplication unit of the A + T-rich region. In addition, the intergenic spacer between *trnS2* and *ND1* was found in all sequenced mantodean mitogenomes, ranging from 17 bp to 37 bp. Alignments of all these intergenic spacer sequences showed a 7 bp highly conserved motif (WTACTTA) which could be considered as the binding site of the transcription termination factor (DmTTF).

**Figure 3:**
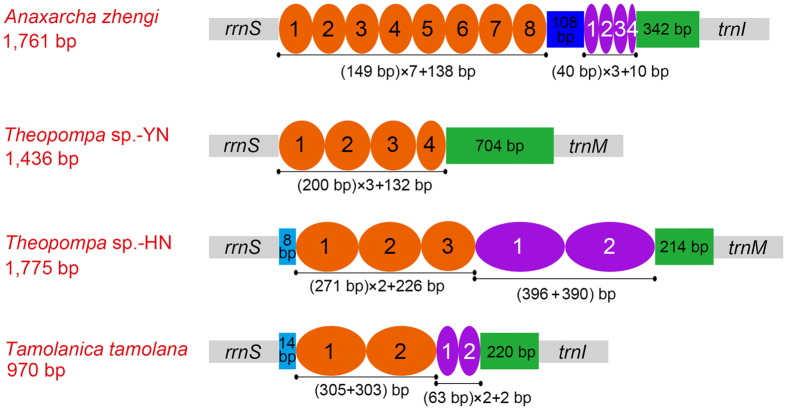
Organization of the A + T-rich region of *Anaxarcha zhengi, Tamolanica tamolana, Theopompa* sp.-YN and *Theopompa* sp.-HN mitochondrial genomes. Oval with different color indicates tandem repeat sequence. Colored box shows the non-repeat region.

The A + T-rich region known as the control region for insect mitogenome is the largest non-coding region in all mantodean mitogenomes. The entire A + T-rich regions have distinguished differences in size, which range from 639 bp in *M. religiosa* to 1,775 bp in *Theopompa* sp.-HN. Four of comparatively larger sequences contain larger tandem repeats (Fig. 3) which are main factor for variation in the length. In *Theopompa* sp.-YN, only one tandem repeat unit with 200 bp extends three identical copies with a partial fourth. While the rest three species (*A. zhengi, T. tamolana* and *Theopompa* sp.-HN) include two different types of tandem repeats. Furthermore, plenty of microsatellite-like elements present in mantodean A + T-rich regions.

### tRNA duplication, reassignment and rearrangements

Mitochondrial tRNA of Mantodea exhibits high evolutionary diversity including duplication, reassignment and rearrangement. Although the extra gene copy is uncommon in animal mitogenome, four of eight praying mantis mitogenomes contain multiple identical tRNA: two *trnR* in *M. religiosa*, three *trnR* in *C. gemmata*, eight *trnR* in *Theopompa* sp.-HN, and six *trnR* and five *trnW*_2_ (excluding *trnW*_1_) in *Statilia* sp. The *trnW*_1_ and *trnW*_2_ of *Statilia* sp. hold the same anticodon but show the low sequence similarity. The obvious sequence similarity of *trnW*_2_ and *tnrR* (Fig. 2C) indicates the duplicate *trnR* underwent a gene recruitment process, namely *trnR* converted to *trnW* by point mutation at the third anticodon position from triplet “TCG” to “TCA”. These uncommon changes contribute to the variable gene content and order in Mantodea mitogenomes. In three Liturgusidae species, *trnM* has moved into the position downstream of the A + T-rich region. The translocation of *trnM* poses a new gene order of this tRNA cluster (*trnM*-*trnI*-*trnQ*). Another novel gene order is present in tRNA cluster between *ND3* and *ND5*. Duplicate *trnR* and *trnW*_2_ alternately occur between *trnA* and *trnN* and generate a unique gene order [*ND3*-*trnA*-(*trnR*)_2_-*trnW*-(*trnR*)_2_-(*trnW*)_2_-(*trnR*-*trnW*)_2_-*trnN*-*trnS*-*trnE*-*trnF*-*ND5*] for this tRNA cluster.

### Phylogenetic analyses

**Figure 4:**
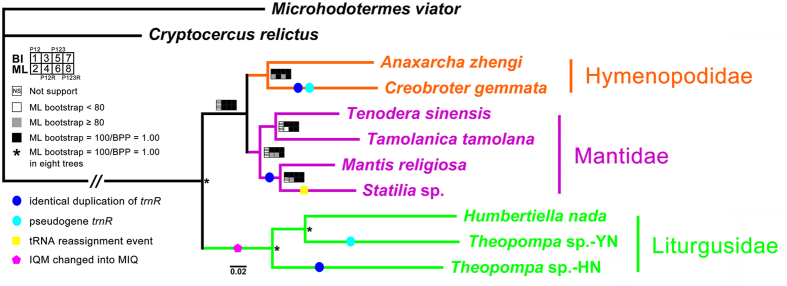
Phylogenetic relationship among nine praying mantises inferred from mitogenome data using BI and ML analyses. Asterisk indicate Bayesian posterior probabilities (BPP) = 1.00 and ML bootstrap = 100 in all Phylogenetic analysis. Squares at the nodes correspond to BPP for 1 (PCG12), 3 (PCG12R), 5 (PCG123), and 7 (PCG123R) and ML bootstrap support values in percentages for 2 (PCG12), 4 (PCG12R), 6 (PCG123) and 8 (PCG123R). NS: not support; white square: ML bootstrap values <80; gray square: ML bootstrap values ≥80; black square: ML bootstrap values = 100 and BPP = 1.00.

The phylogenetic relationships inferred from maximum likelihood (ML) and Bayesian inference (BI) analyses using three datasets (PCG123, PCG123R, and PCG12R) share similar topologies (Fig. 4). Our analyses based on datasets with all mitochondrial genes show a higher branch support values than PCGs dataset. Not surprisingly, Liturgusidae diverged basally from the rest of the species^24^, and all three families (Liturgusidae, Hymenopodidae, and Mantidae) form a monophyly respectively with strong support. Consistent with previous results, both Mantini species *Statilia* sp. and *M. religiosa* share a closer relationship within the Mantidae, and the other two species *T. tamolana* (Paramantini) and *T. sinensis* (Polyspilotini) cluster together as a sister group to the Mantini. While within the clade of Liturgusidae, *Theopompa* is unexpectedly identified as a non-monophyly group with topology ((*Humbertiella nada* + *Theopompa* sp.-YN) + *Theopompa* sp.-HN). The results of PCG12 dataset show different topologies for Mantidae but with low support values (ML bootstrap <50).

## Discussion

Mantodean mitogenomes show numbers of common features observed in other insects such as AT-biased base composition and codon usage. Compared within sequenced dictyopteran mitogenomes, the overall A + T contents of mantodean mitogenomes (70.1–77.8%) are comparable to those of Blattodea (72.0–76.0%)^25^,^26^ but relatively higher than those of Isoptera (63.4–71.4%)^27^. The length of mantodean coding region without duplicate genes is similar to that of other dictyopterans. The intergenic spacers are frequently found in mantodean mitogenomes contained pseudogenes or gene rearrangements, while most dictyopterans with ancestral gene arrangment present more compact mitogenome architecture. The A + T-rich region of mantodean mitogenomes also reflects the general characteristics for dictyopterans including tandem repeat sequences, microsatellite-like elements and poly-T structure. Although several fragments with relatively high similarity were found among close relative species, the conserved blocks could not be identified through all dictyopterans.

Gene duplication generally together with rearrangement occurs in the metazoan mitogenome, and the redundant gene copy might be rapidly deleted or pseudogenized with mutations under strong selective pressure^23^. Although the extra gene copy is uncommon in the metazoan mitogenome, five of eight praying mantis mitogenomes possess the duplication of tRNA. Slipped-strand mispairing could give rise to these repeated *trnR* during mitogenome replication^28^. The identical *trnR* gene sequences demonstrate that all of them are functional *trnR* and the duplicate is a very recent event. In fact, *Theopompa* sp.-YN and *C. gemmata* harbor another pseudogene *trnR* between *trnA* and *trnR*, and *trnR* and *trnN* respectively (Fig. 1). The pseudogene *trnR* of *C. gemmata* has a truncated and mutated 3′ end (acceptor stem secondary structure is absent), while mutations distribute through the degenerated *trnR* in *Theopompa* sp.-YN (Fig. 2B). The physical positions of two pseudogene *trnR* indicate that degeneration can occur in original and new copy of *trnR*, and both of two *trnR* for *Theopompa* sp.-YN may be functional genes before degeneration.

**Figure 5:**
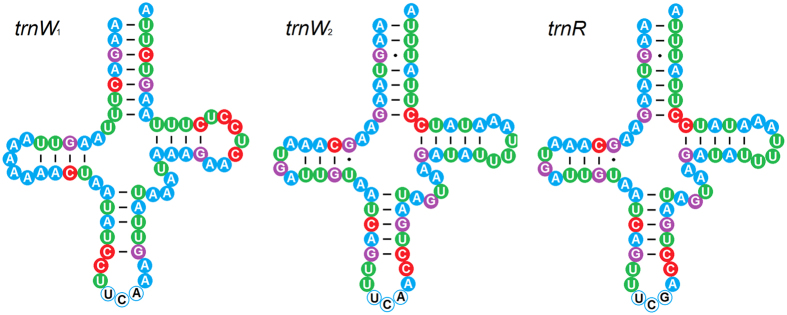
Inferred secondary structure of *trnR* and two *trnW* genes for *Statilia* sp.

Gene duplication usually provides important source of material for formation of evolutionarily related genes with a specific function^29^. As the products of gene duplication, some copies function in the original role, while the others could serve a new function. In *Statilia* sp., two non-homologous sequences encode Tryptophan tRNA genes with same anticodon. The *trnW*_2_ is reassigned from *trnR* by point mutation at anticodon. Although two functional *trnW* is uncommon in animal mitogenomes, the stable secondary structure identical with full functional *trnR* implies potential function for *trnW*_2_ (Fig. 5). Remolding tRNA with only an anticodon point mutation may replace the role of a second disabled tRNA^30^. However, a single point mutation at the anticodon without any post-transcriptional modifications can not change the original advanced structure and identity elements which influence the recognition and interaction between tRNA and aminoacyl-tRNA synthetase. The first base pair A_1_-U_72_ (T_72_ at the DNA level) in acceptor stem and G_73_ are confirmed as the identity elements on *trnW* for bacterial tryptophanyl-tRNA synthetase^31^,^32^,^33^,^34^,^35^, however, unexpectedly both *trnW*_1_ and *trnW*_2_ harbor the same nucleotides for identity elements. Consequently, we cannot rule out the possibility that *trnW*_2_ participates in interactions, in particular to the coexistence with clear functional *trnW*_1_. Gene recruitment is an important mechanism for tRNA evolution, while the direct evidence of recent gene recruitment are detected in limited organisms such as *Amoebidium parasiticum* (Ichthyophonida), *Axinella corrugata* (Demospongiae), *Crassostrea* sp. and *Pinctada maxima* (Bivalvia), and *Tropiocolotes tripolitanus* (Gekkonidae)^36^,^37^,^38^,^39^. Most of mitochondrial tRNA recruitments happen in regular circular mitogenomes, the exception being some organism with linear chromosomes, where these recruitment features of *Statilia* sp. and *T. tripolitanus* provide evidences for verifying that the higher frequency of tRNA gene duplication would facilitate the process of gene recruitment.

**Figure 6:**
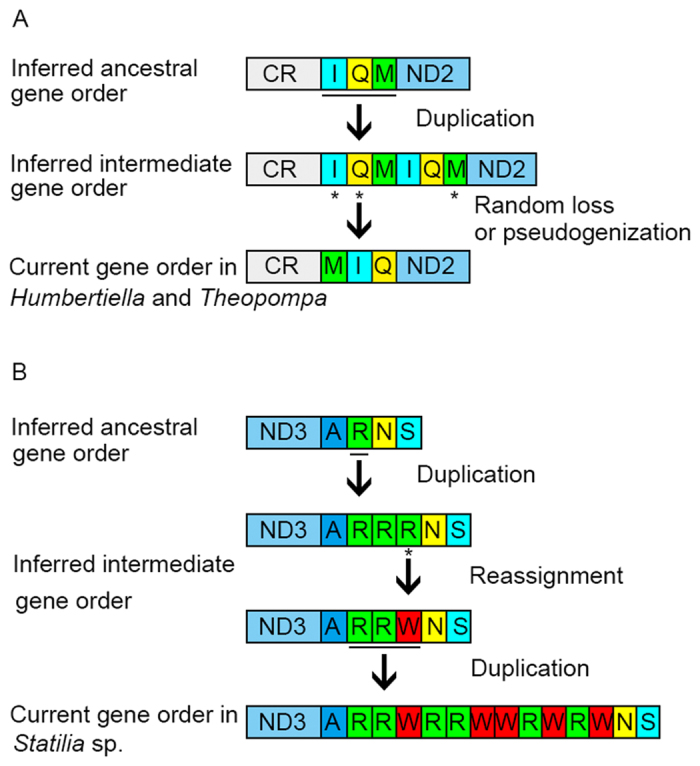
Putative mechanism of mitochondrial gene rearrangements occurring in *Humbertiella nada, Theopompa* spp. and *Statilia* sp.

All available mitogenome organizations of Mantodea are shown in Fig. 1. Only two match the typical ancestral pancrustacean gene order when considering the multiple gene copies of *trnR*. The new gene orders of three Liturgusidae species and *Statilia* sp. are firstly reported in Dictyoptera which indicate Mantodea could no longer be treated as a lineage with low gene rearrangements. For the gene rearrangement of three Liturgusidae species, tandem duplication-random loss (TDRL) model is a plausible mechanism which has been proposed to explain the similar rearrangements in other insects^40^,^41^. We assume that the original gene order of this region is *trnI*-*trnQ*-*trnM*. The tandem duplication of *trnI*-*trnQ*-*trnM* was followed by random deletion of *trnI*-*trnQ* in first set and *trnM* in seconded set (Fig. 6A). In fact, the pseudogene remnant of *trnM* was confidently identified in intergenic spacer between *trnQ* and *ND2* (Fig. 2A). Although mutations indicate *trnM* pseudogene could not fold into stable secondary structure (also including mutations of anticodon), the high level of sequence similarity for alignments of functional and pseudogenes *trnM* from these species respectively suggests that each of them is homologues.

Even though various gene orders of the tRNA cluster between *ND3* and *ND5* were found in a number of insects^42^, the new tRNA gene arrangement in *Statilia* sp. [*trnA*-(*trnR*)_2_-*trnW*-(*trnR*)_2_-(*trnW*)_2_-(*trnR*-*trnW*)_2_-*trnN*] is novel for all sequenced pancrustacea. The unusual and extra *trnR* and *trnW* were generated by tRNA duplication and reassignment. Actually, *trnR* and *trnW* constantly alternated with different copy number indicating that the present gene order was derived from large duplication (might include deletion and/or other process) after reassignment for specific duplicate tRNA (Fig. 6B). We prefer this mechanism considering that reassignment is a rare event within the insect mitogenome, whereas gene duplication occurs more frequently in mitogenome. Moreover the mechanism mentioned above possesses more parsimonious scenario than multiple *trnR* duplication followed by random reassignment for several *trnR*.

Codon usage and tRNA content suffer the co-adaptation evolution in *Escherichia coli, Saccharomyces cerevisiae* and *Caenorhabditis elegans* genomes^43^,^44^,^45^ suggesting the strong correlation between the number of tRNA genes and codon usage. And all the isoaccepting tRNAs corresponding to preferential codons have the highest gene copy number. Recent studies proposed that change of tRNA gene content didn’t influence codon usage bias within vertebrate mitogenomes^9^,^39^. Among invertebrates, large duplications of tRNA gene is exceedingly rare, especially for Insecta. Large duplicate tRNA genes in Mantodea mitogenome provide the chance to investigate the relationship between tRNA gene number and codon usage. The frequencies of codon CGA corresponding *trnR* genes duplication in *Statilia* sp., *M. religiosa, C. gemmata, Theopompa* sp.-HN do not change compared to those of other mantises (Table 2). The use of overall Arginine codons is also stable for all analytical mantodean mitogenomes. In addition, a single *trnW* always decode two codon (UGA and UGG) for mitochondrial protein synthesis in invertebrate mitogenome. However, one *trnW*_1_ and five *trnW*_2_ appear in *Statilia* sp. mitogenome. Despite with five extra *trnW*_2_, the codon use UGA in *Statilia* sp. does not significantly differ from other mantises, and the relative frequency of UGA versus UGG is similar to other mantises. Moreover statistical findings in Table 2 do not show evidence for total Tryptophan codons use change (UGA + UGG). All evidence is indicative of increased tRNA gene copy does not change codon usage bias.

Recently, several molecular studies rejected the monophyly for several families of Mantodea^24^,^46^. Combining our results with previously reported phylogenetic relationships within Mantodea^24^, the common trait of ‘*trnM*-*trnI*-*trnQ*’ for these three species may derive from the ancestor of the Asian bark mantis rather than Liturgusidae, and this feature may also be found in other closely related species and genera. But the novel tRNA gene order in *Statilia* sp. underwent relatively recent multiple changes, implying this gene rearrangement characteristic may be less conserved and just an independent event for specific taxa. Gene order be regularly regarded as useful marker for phylogenetic analysis especially at higher taxonomic levels, but this characteristic of Mantodea is not appropriate for analysis at least in family-level. Another rare tRNA reassignment feature only happened in *Statilia* sp. mitogenome but not in other species with dupilicate *trnR*, although the high level of tRNA gene duplication may facilitate the process of gene recruitment. In animal mitogenomes, gene duplications are recognized as systematic or individual evolutionary event for different lineage. With respect to our findings that the duplication of tRNA occurs in five of nine mantis, including all three families, we cannot immediately confirm this duplication reflects phylogenetic information based on limited samples. But this feature might be helpful for studying lower level relationships.

In summary, we determined eight new mitogenomes of Mantodea including the first representatives from Hymenopodidae and Liturgusidae. These mitogenomes show several unusual features of genomic organization including gene rearrangement, gene duplication and gene reassignment. Comparative analysis of mitogenome sequences involving the changed gene order indicate that tandem duplication-random loss and duplication-reassignment-duplication are the persuasive mechanism for gene rearrangements in Asian bark mantis and *Statilia* sp. respectively. Abundant duplicate tRNA genes do not influence codon usage suggesting the absence of correlation between tRNA gene number and codon usage. In addition, tRNA reassignment by point mutation of anticodon generates an additional *trnW*_2_ in *Statilia* sp., which is really rare in Insecta. And all these various variations of tRNA were considered as potential markers for investigation of lower level relationships. With evolutionary diversity of tRNA, this enigmatic lineage, Mantodea, could expand the understanding of evolution for mitochondrial tRNA and gene order with more samples.

## Methods

### Mitochondrial Genome Sequencing

Eight new praying mantises mitochondrial genomes were obtained from a single specimen respectively (Supplementary Table S10) with overlapping PCR fragments and primer walking. DNA was extracted using the TIANamp Micro DNA Kit (Tiangen Biotech, Beijing, China) according to the manufacturer’s protocol. Universal primers^47^ and specific primers used to amplify mitochondrial genomes are listed in Supplementary Table S11. The overlapping fragments were amplified using FastPfu Fly DNA Polymerase (TransGen Biotech, Beijing, China) with the following cycling conditions: an initial denaturation for 1 min at 93 °C, followed by 35 cycles of 15 sec at 92 °C, 1 min at 45–57 °C, 2–7 min at 72 °C, and final extension of 10 min at 72 °C. After purification with PCR Purification Kit (Sangon Biotech, Shanghai, China), all PCR products were sequenced directly with the PCR primers and internal primers generated by primer walking.

### Genome annotation and sequence analyses

Contiguous sequence fragments were assembled using Staden Package v1.7.0^48^. Protein coding genes (PCGs) and ribosomal RNA (rRNA) genes were identified based on homologous regions of other insects using the Clustal X^49^,^50^. Transfer RNAs (tRNA) and their potential cloverleaf structures were identified by tRNAscan-SE 1.21^51^. The tRNAs, which were not detected by tRNA scan-SE v1.21, were identified by comparing the sequence to *Tamolanica tamolana* (GenBank accession number DQ241797). Tandem Repeat Finder v4.07 was used to identify tandem repeats in non-coding regions^52^. The base composition and codon usage were calculated with MEGA v5.1^53^. All these new mitogenomes of Mantodea have been deposited at GenBank under the accessions KU201313–KU201320.

### Phylogenetic analyses

To infer the phylogenetic relationships among sequenced mantises (*Microhodotermes viator* with GenBank accession number NC_018122 and *Cryptocercus relictus* with GenBank accession number NC_018132 as outgroup^54^), Four datasets (PCG123: 13 PCGs including all codon positions; PCG123R: two rRNAs, 22 tRNAs and 13PCGs including all codon positions; PCG12: 13 PCGs without third codon positions; PCG12R: two rRNAs, 22 tRNAs and 13PCGs without third codon positions) were used to draw the maximum likelihood (ML) and Bayesian inference (BI) phylogeny with the partitioning scheme of rRNA, tRNA and each codon for PCGs (merged codon positions across genes formed three independent codon subsets) to PCG123R and PCG12R, and codon-based partitions to PCG123 and PCG12^55^. We extracted and translated all 13 PCGs using the invertebrate mitochondrial genetic code. Then, the inferred amino acid sequences, rRNA and tRNA genes were individually aligned with Clustal X and ambiguously aligned regions were removed with Gblocks^56^. The best-fit model (GTR + Γ + I) for each partition was selected using Akaike information criterion in jModelTest^57^. For ML analyses implemented in RAxML ver.7.2.8^58^, bootstrap analysis was performed with 1,000 replicates. For BI analyses implemented in MrBayes ver.3.1.2^59^, two sets of four chains were allowed to run simultaneously for 1,000,000 generations. Each set was sampled every 100 generations with a burn-in of 25%.

## Additional Information

**How to cite this article**: Ye, F. *et al*. Mitochondrial genomes of praying mantises (Dictyoptera, Mantodea): rearrangement, duplication, and reassignment of tRNA genes. *Sci. Rep.* **6**, 25634; doi: 10.1038/srep25634 (2016).

## Supplementary Material

Supplementary Information

## Figures and Tables

**Table 1:** Base composition of mantodean mitochondrial genomes.

Family	Species	Whole genome	Protein-coding genes	rRNA genes	tRNA genes	A + T-rich region
Hymenopodidae	*Anaxarcha zhengi*	77.8	76.9	80.2	77.4	79.3
	*Creobroter gemmata*	76.0	75.4	77.4	75.2	79.9
Mantidae	*Tenodera sinensis*	75.5	74.7	77.8	75.5	78.9
	*Tamolanica tamolana*	75.3	74.7	77.0	76.5	74.7
	*Mantis religiosa*	76.7	76.1	77.9	75.8	81.1
	*Statilia* sp.	75.3	74.7	77.6	75.4	76.2
Liturgusidae	*Humbertiella nada*	70.1	68.8	73.1	73.1	73.7
	*Theopompa* sp.-YN	70.7	69.8	74.4	72.7	70.8
	*Theopompa* sp.-HN	71.7	70.6	74.4	75.5	71.2

**Table 2:** Codon usage of codons for Arginine and Tryptophan.

Species	Codons for Arginine					Codons for Tryptophan		
	CGA	CGU	CGC	CGG	Total	UGA	UGG	Total
*Anaxarcha zhengi*	30	23	1	2	56	98	4	102
*Creobroter gemmata*	31	17	1	8	57	97	7	104
*Tenodera sinensis*	36	19	1	2	58	96	8	104
*Tamolanica tamolana*	32	21	2	2	57	101	1	102
*Mantis religiosa*	31	24	0	2	57	98	6	104
*Statilia* sp.	31	18	6	2	57	93	11	104
*Humbertiella nada*	29	16	4	9	58	94	14	108
*Theopompa* sp.-YN	29	15	2	9	55	88	17	105
*Theopompa* sp.-HN	30	17	2	7	56	91	19	110
Average of nine species	31.0	18.9	2.1	4.8	56.8	95.1	9.7	104.8
