# Supplementary material for: Mitochondrial genomes of praying mantises (Dictyoptera, Mantodea): rearrangement, duplication, and reassignment of tRNA genes
Source: Sci Rep. 2016 May 9;6:25634. doi: 10.1038/srep25634 (PMC4860592; doi:10.1038/srep25634)
Supplement: Supplementary Information [file srep25634-s1.pdf]

Mitochondrial genomes of praying mantises (Dictyoptera, Mantodea): rearrangement, duplication, and reassignment of tRNA genes

Fei Ye, Xu-e Lan, Wen-bo Zhu and Ping You*

Co-Innovation Center for Qinba Regions' Sustainable Development, College of Life Science, Shaanxi Normal University, Xi'an, 710062, China

* Corresponding author: youping@snnu.edu.cn

Table S1. The organization of the mitochondrial genome of *Anaxarcha zhengi*

Gene (region)	Strand	Position	Codon		Anticodon
			Start	Stop	
trnI	J	1-66			GAT
trnQ	N	78-147			TTG
trnM	J	147-214			CAT
ND2	J	215-1242	ATG	TA	
trnW	J	1243-1307			TCA
trnC	N	1300-1364			GCA
trnY	N	1374-1438			GTA
COI	J	1459-2997	TTG	TAA	
trnL^{UUR}	J	3011-3075			TAA
COII	J	3079-3766	ATG	T	
trnK	J	3767-3838			CTT
trnD	J	3839-3903			GTC
ATP8	J	3904-4062	ATC	TAA	
ATP6	J	4056-4736	ATG	TAA	
COIII	J	4736-5524	ATG	TAA	
trnG	J	5550-5613			TCC
ND3	J	5614-5967	ATT	TAA	
trnA	J	5974-6041			TGC
trnR	J	6046-6111			TCG
trnN	J	6120-6185			GTT
trnS^{AGN}	J	6186-6252			GCT
trnE	J	6252-6317			TTC
trnF	N	6318-6381			GAA
ND5	N	6382-8104	ATG	T	
trnH	N	8105-8168			GTG
ND4	N	8173-9510	ATG	TAA	
ND4L	N	9504-9785	ATG	TAA	
trnT	J	9789-9858			TGT
trnP	N	9859-9923			TGG
ND6	J	9926-10429	ATT	TAA	
CytB	J	10429-11565	ATG	TAA	
trnS^{UCN}	J	11575-11645			TGA
ND1	N	11679-12617	ATA	TAA	
trnL^{CUN}	N	12621-12685			TAG
rrnL	N	12686-13995			
trnV	N	13996-14064			TAC
rrnS	N	14065-14859			
A+T-rich region	—	14860-16620			

Table S2. The organization of the mitochondrial genome of *Creobroter gemmata*

Gene (region)	Strand	Position	Codon		Anticodon
			Start	Stop	
trnI	J	1-66			GAT
trnQ	N	67-136			TTG
trnM	J	136-202			CAT
ND2	J	203-1231	ATG	TAA	
trnW	J	1236-1302			TCA
trnC	N	1295-1360			GCA
trnY	N	1376-1435			GTA
COI	J	1441-2976	TTG	TAA	
trnL^{UUR}	J	2978-3043			TAA
COII	J	3045-3732	ATG	T	
trnK	J	3733-3804			CTT
trnD	J	3805-3870			GTC
ATP8	J	3871-4029	ATT	TAA	
ATP6	J	4023-4703	GTG	TAA	
COIII	J	4704-5492	ATG	TAA	
trnG	J	5496-5564			TCC
ND3	J	5565-5918	ATT	TAA	
trnA	J	5930-5994			TGC
trnR	J	5999-6063			TCG
trnR	J	6073-6137			TCG
trnR	J	6147-6211			TCG
trnN	J	6280-6345			GTT
trnS^{AGN}	J	6345-6418			GCT
trnE	J	6431-6497			TTC
trnF	N	6496-6560			GAA
ND5	N	6561-8283	ATA	T	
trnH	N	8287-8350			GTG
ND4	N	8354-9691	ATG	TAA	
ND4L	N	9685-9966	ATG	TAA	
trnT	J	9972-10035			TGT
trnP	N	10036-10099			TGG
ND6	J	10102-10605	ATT	TAA	
CytB	J	10605-11738	ATG	TAA	
trnS^{UCN}	J	11740-11809			TGA
ND1	N	11830-12762	ATG	TAA	
trnL^{CUN}	N	12765-12832			TAG
rrnL	N	12833-14154			
trnV	N	14155-14225			TAC
rrnS	N	14226-15008			
A+T-rich region	—	15009-15716			

Table S3. The organization of the mitochondrial genome of *Mantis religiosa*

Gene (region)	Strand	Position	Codon		Anticodon
			Start	Stop	
trnI	J	1-67			GAT
trnQ	N	74-143			TTG
trnM	J	143-208			CAT
ND2	J	209-1235	ATG	T	
trnW	J	1236-1304			TCA
trnC	N	1297-1360			GCA
trnY	N	1363-1430			GTA
COI	J	1435-2973	TTG	TAA	
trnL^{UUR}	J	2983-3048			TAA
COII	J	3053-3737	ATG	T	
trnK	J	3738-3809			CTT
trnD	J	3812-3879			GTC
ATP8	J	3880-4038	ATT	TAA	
ATP6	J	4032-4712	ATG	TAA	
COIII	J	4715-5506	ATG	TAA	
trnG	J	5509-5572			TCC
ND3	J	5573-5926	ATT	TAA	
trnA	J	5928-5992			TGC
trnR	J	6001-6068			TCG
trnR	J	6088-6155			TCG
trnN	J	6156-6221			GTT
trnS^{AGN}	J	6222-6288			GCT
trnE	J	6295-6360			TTC
trnF	N	6361-6426			GAA
ND5	N	6427-8146	ATA	T	
trnH	N	8150-8214			GTG
ND4	N	8218-9555	ATG	TAA	
ND4L	N	9549-9830	ATG	TAA	
trnT	J	9834-9897			TGT
trnP	N	9898-9960			TGG
ND6	J	9963-10466	ATT	TAA	
CytB	J	10478-11614	ATG	TAA	
trnS^{UCN}	J	11620-11689			TGA
ND1	N	11711-12646	ATG	TAA	
trnL^{CUN}	N	12648-12715			TAG
rrnL	N	12716-14032			
trnV	N	14033-14102			TAC
rrnS	N	14103-14895			
A+T-rich region	—	14896-15534			

Table S4. The organization of the mitochondrial genome of *Tenodera sinensis*

Gene (region)	Strand	Position	Codon		Anticodon
			Start	Stop	
trnI	J	1-67			GAT
trnQ	N	80-148			TTG
trnM	J	148-215			CAT
ND2	J	216-1242	ATG	T	
trnW	J	1243-1312			TCA
trnC	N	1305-1368			GCA
trnY	N	1375-1444			GTA
COI	J	1459-2997	ATT	TAA	
trnL^{UUR}	J	3007-3072			TAA
COII	J	3078-3762	ATG	T	
trnK	J	3763-3834			CTT
trnD	J	3836-3901			GTC
ATP8	J	3902-4060	ATC	TAA	
ATP6	J	4054-4734	ATG	TAA	
COIII	J	4734-5525	ATA	TAA	
trnG	J	5528-5592			TCC
ND3	J	5593-5946	ATT	TAA	
trnA	J	5949-6013			TGC
trnR	J	6018-6085			TCG
trnN	J	6088-6153			GTT
trnS^{AGN}	J	6154-6220			GCT
trnE	J	6226-6292			TTC
trnF	N	6293-6357			GAA
ND5	N	6358-8077	ATA	T	
trnH	N	8081-8145			GTG
ND4	N	8150-9487	ATG	TAA	
ND4L	N	9481-9762	ATG	TAA	
trnT	J	9767-9830			TGT
trnP	N	9831-9894			TGG
ND6	J	9897-10400	ATT	TAA	
CytB	J	10400-11534	ATG	T	
trnS^{UCN}	J	11535-11606			TGA
ND1	N	11626-12561	ATG	TAA	
trnL^{CUN}	N	12563-12630			TAG
rrnL	N	12631-13941			
trnV	N	13942-14013			TAC
rrnS	N	14014-14825			
A+T-rich region	—	14826-15531			

Table S5. The organization of the mitochondrial genome of *Statilia* sp.

Gene (region)	Strand	Position	Codon		Anticodon
			Start	Stop	
trnI	J	1-65			GAT
trnQ	N	73-142			TTG
trnM	J	142-208			CAT
ND2	J	209-1235			
trnW	J	1236-1302			TCA
trnC	N	1295-1358			GCA
trnY	N	1360-1430			GTA
COI	J	1438-2973	TTG	TAA	
$trnL^{UUR}$	J	2982-3046			TAA
COII	J	3051-3735	ATG	T	
trnK	J	3736-3807			CTT
trnD	J	3813-3878			GTC
ATP8	J	3879-4037	ATT	TAA	
ATP6	J	4031-4711	ATG	TAA	
COIII	J	4714-5502	ATG	TAA	
trnG	J	5505-5568			TCC
ND3	J	5569-5922	ATT	TAA	
trnA	J	5945-6009			TGC
trnR	J	6011-6077			TCG
trnR	J	6096-6162			TCG
trnW	J	6181-6247			TCA
trnR	J	6266-6332			TCG
trnR	J	6351-6417			TCG
trnW	J	6436-6502			TCA
trnW	J	6521-6587			TCA
trnR	J	6606-6672			TCG
trnW	J	6691-6757			TCA
trnR	J	6776-6842			TCG
trnW	J	6861-6927			TCA
trnN	J	6934-6998			GTT
$trnS^{AGN}$	J	6999-7065			GCT
trnE	J	7070-7133			TTC
trnF	N	7134-7197			GAA
ND5	N	7198-8917	ATA	T	
trnH	N	8921-8985			GTG
ND4	N	8988-10325	ATG	TAA	
ND4L	N	10319-10600	ATG	TAA	
trnT	J	10604-10667			TGT
trnP	N	10668-10730			TGG
ND6	J	10733-11236	ATT	TAA	
CytB	J	11236-12372	ATG	TAA	
$trnS^{UCN}$	J	12379-12448			TGA
ND1	N	12470-13405	ATG	TAA	
$trnL^{CUN}$	N	13407-13474			TAG
rrnL	N	13475-14785			
trnV	N	14786-14854			TAC
rrnS	N	14855-15642			
A+T-rich region	—	15643-16294			

Table S6. The organization of the mitochondrial genome of *Humbertiella nada*

Gene (region)	Strand	Position	Codon		Anticodon
			Start	Stop	
trnM	J	1-68			CAT
trnI	J	207-272			GAT
trnQ	N	282-351			TTG
ND2	J	416-1444	ATT	TAA	
trnW	J	1464-1530			TCA
trnC	N	1523-1587			GCA
trnY	N	1589-1653			GTA
COI	J	1653-3188	CTG	TAA	
trnL^{UUR}	J	3194-3258			TAA
COII	J	3259-3943			
trnK	J	3944-4014			CTT
trnD	J	4014-4078			GTC
ATP8	J	4079-4240	ATT	TAA	
ATP6	J	4234-4914	ATG	TAA	
COIII	J	4914-5700	ATG	T	
trnG	J	5701-5762			TCC
ND3	J	5763-6114	ATT	T	
trnA	J	6115-6178			TGC
trnR	J	6183-6251			TCG
trnN	J	6265-6330			GTT
trnS^{AGN}	J	6331-6397			GCT
trnE	J	6402-6467			TTC
trnF	N	6466-6529			GAA
ND5	N	6532-8252	ATG	TA	
trnH	N	8253-8315			GTG
ND4	N	8326-9663	ATG	TAA	
ND4L	N	9657-9938	ATG	TAA	
trnT	J	9945-10014			TGT
trnP	N	10015-10078			TGG
ND6	J	10081-10587	ATT	TAA	
CytB	J	10587-11722	ATG	TA	
trnS^{UCN}	J	11728-11799			TGA
ND1	N	11817-12749	ATG	TAA	
trnL^{CUN}	N	12751-12818			TAG
rrnL	N	12819-14137			
trnV	N	14138-14206			TAC
rrnS	N	14207-15000			
A+T-rich region	—	15001-15866			

Table S7. The organization of the mitochondrial genome of *Theopompa* sp.-YN

Gene (region)	Strand	Position	Codon		Anticodon
			Start	Stop	
trnM	J	1-68			CAT
trnI	J	198-264			GAT
trnQ	N	255-323			TTG
ND2	J	392-1423	ATT	TAA	
trnW	J	1427-1492			TCA
trnC	N	1485-1546			GCA
trnY	N	1547-1612			GTA
COI	J	1612-3147	CTG	TAG	
trnL^{UUR}	J	3155-3218			TAA
COII	J	3220-3904	ATG	T	
trnK	J	3905-3974			CTT
trnD	J	3978-4041			GTC
ATP8	J	4042-4200	ATT	TAA	
ATP6	J	4194-4874	ATG	TAA	
COIII	J	4874-5660	ATG	T	
trnG	J	5661-5722			TCC
ND3	J	5723-6074	ATT	T	
trnA	J	6075-6137			TGC
trnR	J	6207-6270			TCG
trnN	J	6298-6361			GTT
trnS^{AGN}	J	6362-6428			GCT
trnE	J	6429-6491			TTC
trnF	N	6492-6555			GAA
ND5	N	6558-8278	ATG	TA	
trnH	N	8279-8341			GTG
ND4	N	8351-9688	ATG	TAA	
ND4L	N	9682-9963	ATG	TAA	
trnT	J	9970-10032			TGT
trnP	N	10033-10095			TGG
ND6	J	10098-10601	ATT	TAA	
CytB	J	10601-11735	ATG	T	
trnS^{UCN}	J	11736-11807			TGA
ND1	N	11827-12762	ATG	TAA	
trnL^{CUN}	N	12764-12830			TAG
rrnL	N	12831-14140			
trnV	N	14141-14210			TAC
rrnS	N	14211-14995			
A+T-rich region	—	14996-16431			

Table S8. The organization of the mitochondrial genome of *Theopompa* sp.-HN

Gene (region)	Strand	Position	Codon		Anticodon
			Start	Stop	
trnI	J	1-61			GAT
trnQ	N	59-127			TTG
ND2	J	187-1215	ATT	TAA	
trnW	J	1219-1284			TCA
trnC	N	1277-1338			GCA
trnY	N	1339-1403			GTA
COI	J	1403-2938	CTG	TAA	
trnL^{UUR}	J	2945-3008			TAA
COII	J	3011-3695	ATG	T	
trnK	J	3696-3767			CTT
trnD	J	3768-3831			GTC
ATP8	J	3832-3990	ATT	TAA	
ATP6	J	3984-4664	ATG	TAA	
COIII	J	4664-5450	ATG	T	
trnG	J	5451-5512			TCC
ND3	J	5513-5864	ATC	T	
trnA	J	5865-5928			TGC
trnR	J	5933-5998			TCG
trnR	J	6027-6092			TCG
trnR	J	6121-6186			TCG
trnR	J	6215-6280			TCG
trnR	J	6309-6374			TCG
trnR	J	6403-6468			TCG
trnR	J	6470-6535			TCG
trnR	J	6564-6629			TCG
trnR	J	6658-6723			TCG
trnR	J	6752-6817			TCG
trnN	J	6839-6903			GTT
trnS^{AGN}	J	6904-6970			GCT
trnE	J	6971-7035			TTC
trnF	N	7037-7100			GAA
ND5	N	7101-8823	ATG	T	
trnH	N	8824-8886			GTG
ND4	N	8896-10233	ATG	TAA	
ND4L	N	10227-10508	ATG	TAA	
trnT	J	10513-10576			TGT
trnP	N	10577-10640			TGG
ND6	J	10643-11146	ATC	TAA	
CytB	J	11146-12283	ATG	T	
trnS^{UCN}	J	12284-12355			TGA
ND1	N	12374-13306	ATG	TAA	
trnL^{CUN}	N	13308-13373			TAG
rrnL	N	13374-14689			
trnV	N	14690-14758			TAC
rrnS	N	14759-15543			
A+T-rich region	—	15544-17318			
trnM	J	17319-17370			CAT

Table S9. Codon usage for13 protein coding genes of nine praying mantises mitochondrial genomes.

Codon(AA)	Number of used codon									Relative synonymous codon usage								
	Az	Cg	Ts	Tt	Mr	Ssp	Hn	TspY	TspH	Az	Cg	Ts	Tt	Mr	Ssp	Hn	TspY	TspH
UUU(F)	299	284	276	287	292	290	229	252	258	1.83	1.76	1.69	1.72	1.8	1.77	1.49	1.61	1.59
UUC(F)	28	38	50	46	33	38	79	61	66	0.17	0.24	0.31	0.28	0.2	0.23	0.51	0.39	0.41
UUA(L)	471	427	417	424	453	420	273	303	307	5.03	4.6	4.42	4.42	4.74	4.5	2.82	3.06	3.32
UUG(L)	22	33	39	45	17	28	56	50	38	0.23	0.36	0.41	0.47	0.18	0.3	0.58	0.5	0.41
CUU(L)	34	46	54	58	66	65	92	107	90	0.36	0.5	0.57	0.61	0.69	0.7	0.95	1.08	0.97
CUC(L)	2	7	10	6	4	12	51	39	35	0.02	0.08	0.11	0.06	0.04	0.13	0.53	0.39	0.38
CUA(L)	31	41	43	40	31	34	91	70	74	0.33	0.44	0.46	0.42	0.32	0.36	0.94	0.71	0.8
CUG(L)	2	3	3	2	3	1	18	26	11	0.02	0.03	0.03	0.02	0.03	0.01	0.19	0.26	0.12
AUU(I)	338	355	315	316	335	332	291	283	283	1.83	1.82	1.8	1.77	1.91	1.87	1.63	1.63	1.56
AUC(I)	32	36	35	42	15	24	66	65	80	0.17	0.18	0.2	0.23	0.09	0.13	0.37	0.37	0.44
AUA(I)	272	262	240	238	243	228	194	192	226	1.84	1.85	1.77	1.82	1.88	1.77	1.61	1.65	1.72
AUG(M)	23	22	31	23	16	30	47	41	37	0.16	0.15	0.23	0.18	0.12	0.23	0.39	0.35	0.28
GUU(V)	77	71	79	88	107	76	86	80	81	1.75	1.63	1.59	1.86	2.21	1.59	1.69	1.52	1.6
GUC(V)	2	5	8	9	4	6	18	12	17	0.05	0.11	0.16	0.19	0.08	0.13	0.35	0.23	0.34
GUA(V)	93	91	105	82	81	101	73	100	93	2.11	2.09	2.11	1.74	1.67	2.12	1.44	1.9	1.84
GUG(V)	4	7	7	10	2	8	26	18	11	0.09	0.16	0.14	0.21	0.04	0.17	0.51	0.34	0.22
UCU(S)	78	87	91	91	99	96	88	94	80	1.9	2.12	2.16	2.19	2.32	2.29	2.01	2.1	1.98
UCC(S)	9	14	15	24	13	23	35	33	40	0.22	0.34	0.36	0.58	0.3	0.55	0.8	0.74	0.99
UCA(S)	127	99	110	94	111	101	103	94	78	3.1	2.41	2.61	2.27	2.6	2.4	2.35	2.1	1.93
UCG(S)	1	8	0	7	1	3	4	7	5	0.02	0.19	0	0.17	0.02	0.07	0.09	0.16	0.12
CCU(P)	76	74	81	71	85	76	71	84	64	2.32	2.16	2.38	2.14	2.48	2.25	2.09	2.42	1.83
CCC(P)	10	15	14	9	11	14	22	18	28	0.31	0.44	0.41	0.27	0.32	0.41	0.65	0.52	0.8
CCA(P)	42	45	39	52	40	43	43	31	42	1.28	1.31	1.15	1.56	1.17	1.27	1.26	0.89	1.2
CCG(P)	3	3	2	1	1	2	0	6	6	0.09	0.09	0.06	0.03	0.03	0.06	0	0.17	0.17
ACU(T)	64	83	80	69	93	82	79	93	86	1.45	1.78	1.71	1.48	1.98	1.74	1.69	1.95	1.82
ACC(T)	6	15	13	23	11	16	36	31	37	0.14	0.32	0.28	0.49	0.23	0.34	0.77	0.65	0.78
ACA(T)	106	87	92	92	83	87	65	65	63	2.4	1.86	1.97	1.98	1.77	1.85	1.39	1.36	1.33
ACG(T)	1	2	2	2	1	3	7	2	3	0.02	0.04	0.04	0.04	0.02	0.06	0.15	0.04	0.06
GCU(A)	82	93	77	70	94	98	93	90	66	1.95	2.23	1.76	1.65	2.15	2.21	2.02	2.12	1.51
GCC(A)	10	14	9	23	7	9	30	27	37	0.24	0.34	0.21	0.54	0.16	0.2	0.65	0.64	0.85
GCA(A)	74	56	82	72	74	67	57	49	63	1.76	1.34	1.87	1.69	1.69	1.51	1.24	1.15	1.44

GCG(A)	2	4	7	5	0	3	4	4	9	0.05	0.1	0.16	0.12	0	0.07	0.09	0.09	0.21
UAU(Y)	179	158	161	165	181	172	130	131	135	1.85	1.78	1.73	1.74	1.89	1.79	1.5	1.58	1.54
UAC(Y)	15	20	25	25	11	20	43	35	40	0.15	0.22	0.27	0.26	0.11	0.21	0.5	0.42	0.46
UAA(*)	0	0	0	0	0	0	0	0	0	0	0	0	0	0	0	0	0	0
UAG(*)	0	0	0	0	0	0	0	0	0	0	0	0	0	0	0	0	0	0
CAU(H)	58	50	54	60	55	58	57	51	52	1.71	1.47	1.54	1.67	1.55	1.61	1.48	1.24	1.41
CAC(H)	10	18	16	12	16	14	20	31	22	0.29	0.53	0.46	0.33	0.45	0.39	0.52	0.76	0.59
CAA(Q)	55	59	59	57	63	60	54	55	64	1.9	1.87	1.84	1.81	1.94	1.9	1.59	1.67	1.91
CAG(Q)	3	4	5	6	2	3	14	11	3	0.1	0.13	0.16	0.19	0.06	0.1	0.41	0.33	0.09
AAU(N)	171	154	159	152	168	167	130	134	127	1.84	1.74	1.78	1.7	1.89	1.8	1.59	1.57	1.49
AAC(N)	15	23	20	27	10	19	34	37	44	0.16	0.26	0.22	0.3	0.11	0.2	0.41	0.43	0.51
AAA(K)	88	88	71	90	78	82	63	67	81	1.81	1.8	1.58	1.91	1.7	1.8	1.48	1.6	1.62
AAG(K)	9	10	19	4	14	9	22	17	19	0.19	0.2	0.42	0.09	0.3	0.2	0.52	0.4	0.38
GAU(D)	61	56	61	56	65	61	47	48	54	1.82	1.62	1.82	1.62	1.94	1.82	1.45	1.48	1.61
GAC(D)	6	13	6	13	2	6	18	17	13	0.18	0.38	0.18	0.38	0.06	0.18	0.55	0.52	0.39
GAA(E)	82	75	76	79	78	72	72	69	73	1.89	1.85	1.85	1.9	1.86	1.73	1.6	1.59	1.7
GAG(E)	5	6	6	4	6	11	18	18	13	0.11	0.15	0.15	0.1	0.14	0.27	0.4	0.41	0.3
UGU(C)	42	46	39	38	39	42	32	35	45	1.87	1.88	1.9	1.81	1.95	1.95	1.56	1.59	1.96
UGC(C)	3	3	2	4	1	1	9	9	1	0.13	0.12	0.1	0.19	0.05	0.05	0.44	0.41	0.04
UGA(W)	98	97	96	101	98	93	94	88	91	1.92	1.87	1.85	1.98	1.88	1.79	1.74	1.68	1.65
UGG(W)	4	7	8	1	6	11	14	17	19	0.08	0.13	0.15	0.02	0.12	0.21	0.26	0.32	0.35
CGU(R)	23	17	19	21	24	18	16	15	17	1.64	1.19	1.31	1.47	1.68	1.26	1.1	1.09	1.21
CGC(R)	1	1	1	2	0	6	4	2	2	0.07	0.07	0.07	0.14	0	0.42	0.28	0.15	0.14
CGA(R)	30	31	36	32	31	31	29	29	30	2.14	2.18	2.48	2.25	2.18	2.18	2	2.11	2.14
CGG(R)	2	8	2	2	2	2	9	9	7	0.14	0.56	0.14	0.14	0.14	0.14	0.62	0.65	0.5
AGU(S)	39	45	33	42	36	30	28	29	36	0.95	1.09	0.78	1.01	0.84	0.71	0.64	0.65	0.89
AGC(S)	2	3	0	4	3	8	15	13	10	0.05	0.07	0	0.1	0.07	0.19	0.34	0.29	0.25
AGA(S)	72	71	88	70	77	75	72	79	74	1.76	1.73	2.09	1.69	1.81	1.79	1.64	1.77	1.83
AGG(S)	0	2	0	0	1	0	6	9	1	0	0.05	0	0	0.02	0	0.14	0.2	0.02
GGU(G)	76	80	91	77	91	73	39	61	54	1.41	1.5	1.66	1.41	1.7	1.34	0.68	1.09	0.99
GGC(G)	6	5	3	5	3	7	18	16	10	0.11	0.09	0.05	0.09	0.06	0.13	0.31	0.29	0.18
GGA(G)	121	112	108	112	107	100	107	92	105	2.25	2.1	1.97	2.06	2	1.83	1.87	1.64	1.92
GGG(G)	12	16	17	24	13	38	65	55	50	0.22	0.3	0.31	0.44	0.24	0.7	1.14	0.98	0.91

Notes: AA: amino acid; Az: *Anaxarcha zhengi*; Cg: *Creobroter gemmata*; Ts: *Tenodera sinensis*; Tt: *Tamolanica tamolana*; Mr: *Mantis religiosa*; Ssp: *Statilia* sp.; Hn: *Humbertiella nada*; TspY: *Theopomp*a sp.-YN; TspH: *Theopompa* sp.-HN.

Table S10. Specimen information and GenBank accession number for sequenced mitogenomes in this study

Family	Species	Locality	accession number
Hymenopodidae	*Anaxarcha zhengi*	Xunyangba, Ningshan, Shannxi	KU201320
	Creobroter gemmata	Yucai, Sanya, Hainan	KU201319
Mantidae	*Tenodera sinensis*	Mulanshan, Wuhan, Hubei	KU201318
	Mantis religiosa	Hequan, Dingbian, Shannxi	KU201317
	Statilia sp.	Yinggeling Nature Reserve, Hainan	KU201316
Liturgusidae	*Humbertiella nada*	Wuzhishan, Hainan	KU201315
	Theopompa sp.-YN	Mengla, Yunnan	KU201314
	Theopompa sp.-HN	Wuzhishan, Hainan	KU201313

Table S11. Primer names and sequences used for the amplification of mitogenomes in this study

Species	Primer	Primer sequence (5' -3')	Gene	Reference
Anaxarcha zhengi	AzLP1F	ATTCACCTCATCCAACCATTC	*rrnL*	This study
	AzLP1R	ATCCTGTTCCTGCACCACTTT	*COI*	This study
	AzLP2F	TTATCCTCCATTATCTGCT	*COI*	This study
	AzLP2R	GCTTTTATTCGATCAACAT	*trnS*	This study
	AzLP3F	TCCAATCTTAATCAAGTCAT	*ND4L*	This study
	AzLP3R	CGGAAGATGTACTTAGAAAGC	*rrnS*	This study
Creobroter gemmata	CgLP1F	AATAAAGTAAAAAAAGCAACACC	*ND1*	This study
	CgLP1R	ATGACCAAAAAATCAAAACAAGT	*COI*	This study
	CgLP2F	ATCAAGAATCATAGGAGCA	*COI*	This study
	CgLP2R	GAAGACATAAGTTAGCAGC	*trnS*	This study
	CgLP3F	TATTGCCTTACTACTACCA	*ND3*	This study
	CgLP3R	CTATAGATAAGGGGTGATT	*ND6*	This study
	CgLP4F	CTGATACAGGAGCCTCTACAT	*ND4*	This study
	CgLP4R	TTTTAGAAGGGATCAACTTTT	*rrnL*	This study
Tenodera sinensis	TsLP1F	AACCATTCATACCAGCCTTC	*rrnL*	This study
	TsLP1R	TGCTTCCTTTTTTCCTCTCT	*COI*	This study
	TsLP2F	GTTATTGTAACTGCTCACG	*COI*	This study
	TsLP2R	TTCGATCAACACATCTTCT	*trnS*	This study
	TsLP3F	CTACCTTTCTCATTACGATTT	*ND3*	This study
	TsLP3R	TTGATGCTAGTCTAGTCACAT	*ND6*	This study
	TsLP4F	AAACCTAAAGCACCCTCACAAAC	*ND4L*	This study
	TsLP4R	CGTAACAAAGTAGGTGTATCGGA	*rrnS*	This study
Mantis religiosa	MrLP1F	AAACCAACCTGGCTCACTCC	*rrnL*	This study
	MrLP1R	TCCAAAAGCTTCCTTCTTAC	*COI*	This study
	MrLP2F	ATTTTATCTTTGGTGCTTG	*COI*	This study
	MrLP2R	TACCTCTATTTTGGTTTCA	*trnN*	This study
	MrLP3F	TGAGCATCATAATAGGGTTG	*ND3*	This study
	MrLP3R	TGTGTTTTACGTAAGGGTTT	*CYTB*	This study
	MrLP4F	TAAATGCTTACGCTTAGAT	*ND4L*	This study
	MrLP4R	AAGGTTTTCATTTACACTG	*trnV*	This study
Statilia sp.	StspLP1F	TAAAATACCTACAAAACCAACT	*ND1*	This study

	StspLP1R	ACTACAAATCCTAAGAAACCAA	*COI*	This study
	StspLP2F	ATTTTACCTTTTTTTTTGC	*COI*	This study
	StspLP2R	AACCTTGATTTCATTCATG	*ND3*	This study
	StspLP3F	TAGATCATCTCGCCTACCA	*ND3*	This study
	StspLP3R	AGTTTCAATAATTCGCACC	*ND4L*	This study
	StspLP4F	ACCCAGAAGAACATAGACCA	*ND4*	This study
	StspLP4R	TATTAAGGGACGAGAAGACC	*rrnL*	This study
Humbertiella nada	HnLP1F	TATCTTAATCCAACATCGAG	*rrnL*	This study
	HnLP1R	TGGTATAAAATAGGGTCTCC	*COI*	This study
	HnLP2F	TTAGAAGAATAGTTGAAAGAGG	*COI*	This study
	HnLP2R	AGGGATAGTAATCCATAGAGTG	*ND5*	This study
	HnLP3F	GCTGATTCTGTGTTTGGAT	*COIII*	This study
	HnLP3R	TATTTGTGGTCTTTGGGTA	*ND4L*	This study
	HnLP4F	AGGTGTAAAAGAGCAGGTA	*ND4*	This study
	HnLP4R	ATAGTTTGGTATTAGTGGA	*rrnL*	This study
Theopompa sp.-YN	ThYNLP1F	CTTGTTTAATTCTTGTCTTCTCGT	*ND2*	This study
	ThYNLP1R	CCTATTTTGGTAAGTTTAGTTGGG	*ND5*	This study
	ThYNLP2F	TTGAAGATCGTGAAAAAAGATC	*ND3*	This study
	ThYNLP2R	AAGAATAGAAAGGTAAGACTGA	*ND6*	This study
	ThYNLP3F	ATAAAGGTAATGAGGCTGTT	*ND4*	This study
	ThYNLP3R	AATAGGAAGAAAGTGAAAGT	*rrnL*	This study
Theopompa sp.-HN	ThHNLP1F	GATTTGACCCTATTTCCTCTTC	*ND3*	This study
	ThHNLP1R	TTTATCCTTTGCTGTATGTGAG	*ND4L*	This study
	ThHNLP2F	ATAGACCCTGACACTGGTGCTT	*ND4*	This study
	ThHNLP2R	TATGAATGGTTGGACGAGGTAG	*rrnL*	This study
Universal	TM-N200	ACCTTTATAARTGGGGTATGARCC	*trnM*	[43]
	C1-J1709	AATTGGWGGWTTYGGAAAYTG	*COI*	[43]
	C1-N2353	GCTCGTGTATCAACGTCTATWCC	*COI*	[43]
	C1-N2776	GGTAATCAGAGTATCGWCGNGG	*COI*	[43]
	C2-N3665	CCACAAATTTCTGAACATTG	*COII*	[43]
	N3-N5731	TTAGGGTCAAATCCRCAYTC	*ND3*	[43]
	TN-J6155	TTTAATTGAARCCAAAAAGAGG	*trnN*	[43]
	N4-J9172	CGCTCAGGYTGRTACCCYCA	*ND4*	[43]
	N4L-N9629	GTTTGTGAGGGWGYTTTRGG	*ND4L*	[43]
	CB-N11010	TATCTACAGCRAATCCYCCYCA	*CytB*	[43]
	LR-J12888	CCGGTCTGAACTCARATCATGTA	*rrnL*	[43]
	SR-J14197	GTACAYCTACTATGTTACGACTT	*rrnS*	[43]
	SR-N14220	ATATGYACAYATCGCCCGTC	*rrnS*	[43]
